# Case in Practice

**Published:** 1894-05

**Authors:** E. W. Stevens

**Affiliations:** Cameron, Mo.


### CASE IN PRACTICE.

CAMERON, MO., March 15, 1894.

TO THE EDITOR:

SIR,—I would like to state a case, and ask a question through the INTERNATIONAL DENTAL JOURNAL.

CASE.—A young lady of twenty-two, has both of her upper lateral incisors turned wrong side out. They are perfectly formed; instead of being crowded, her front teeth all have liberal spaces between; gums perfectly solid and healthy.

*Question.*—Can these teeth be extracted and turned right side out with perfect safety? If so, should the pulp be removed or not?

As a matter of general information, I solicit replies either through the JOURNAL or personally.

Respectfully,

E. W. STEVENS.

